# Supplementary material for: Exercise and/or Genistein Do Not Revert 24-Week High-Fat, High-Sugar Diet-Induced Gut Microbiota Diversity Changes in Male C57BL/6J Adult Mice
Source: Microorganisms. 2022 Nov 10;10(11):2221. doi: 10.3390/microorganisms10112221 (PMC9693056; doi:10.3390/microorganisms10112221)
Supplement: Supplementary file 1 [file microorganisms-10-02221-s001.zip › microorganisms-1974525-supplementary.pdf]

Supplementary tables

Table S1. Jaccard distance matrix pairwise-comparisons between HFD+chow, chow, HFD, HFD+Gen, HFD+Exe, and HFD+Gen+Exe after 24 weeks of six different 24-week dietary and exercise				
**Group 1**	**Group 2**	**Pseudo-F**	**p-value**	**q-value**
HFD+chow	HFD	9.59703999	0.001	0.00107143
HFD+chow	HFD +Gen	9.12753845	0.001	0.00107143
HFD+chow	HFD+Exe	11.0687531	0.001	0.00107143
HFD+chow	HFD+Gen+Exe	8.14320355	0.001	0.00107143
HFD+chow	Chow	4.66159369	0.001	0.00107143
HFD	HFD +Gen	3.34386645	0.001	0.00107143
HFD	HFD+Exe	2.65870412	0.001	0.00107143
HFD	HFD+Gen+Exe	2.43799975	0.002	0.002
HFD	Chow	11.881784	0.001	0.00107143
HFD +Gen	HFD+Exe	3.23250204	0.001	0.00107143
HFD +Gen	HFD+Gen+Exe	2.39218954	0.001	0.00107143
HFD +Gen	Chow	10.6219254	0.001	0.00107143
HFD+Exe	HFD+Gen+Exe	2.59137522	0.001	0.00107143
HFD+Exe	Chow	13.3345591	0.001	0.00107143

Table S2. Unweighted Unifrac pairwise-comparisons between HFD+chow, chow, HFD, HFD+Gen, HFD+Exe, and HFD+Gen+Exe after 24 weeks of six different 24-week dietary and exercise				
**Group 1**	**Group 2**	**Pseudo-F**	**p-value**	**q-value**
HFD+chow	HFD	12.1230352	0.001	0.00125
HFD+chow	HFD +Gen	10.1686063	0.001	0.00125
HFD+chow	HFD+Exe	12.4506619	0.001	0.00125
HFD+chow	HFD+Gen+Exe	9.74305948	0.001	0.00125
HFD+chow	Chow	4.30379276	0.001	0.00125
HFD	HFD +Gen	4.00450051	0.001	0.00125
HFD	HFD+Exe	3.63841065	0.001	0.00125
HFD	HFD+Gen+Exe	2.2794148	0.016	0.016
HFD	Chow	16.4930738	0.001	0.00125
HFD +Gen	HFD+Exe	3.19899435	0.001	0.00125
HFD +Gen	HFD+Gen+Exe	2.86568999	0.008	0.00857143
HFD +Gen	Chow	13.9254802	0.001	0.00125
HFD+Exe	HFD+Gen+Exe	2.64860284	0.003	0.00346154
HFD+Exe	Chow	18.1948234	0.001	0.00125

Table S3. Bray-Curtis distance matrix pairwise-comparisons between HFD+chow, chow, HFD, HFD+Gen, HFD+Exe, and HFD+Gen+Exe after 24 weeks of six different 24-week dietary and exercise

Group 1	Group 2	Pseudo-F	p-value	q-value
HFD+chow	HFD	9.06653468	0.001	0.00166667
HFD+chow	HFD +Gen	8.60757279	0.001	0.00166667
HFD+chow	HFD+Exe	9.17550596	0.001	0.00166667
HFD+chow	HFD+Gen+Exe	6.59896651	0.001	0.00166667
HFD+chow	Chow	4.0343831	0.001	0.00166667
HFD	HFD +Gen	2.45956853	0.027	0.03115385
HFD	HFD+Exe	2.58367891	0.017	0.02125
HFD	HFD+Gen+Exe	1.20595206	0.258	0.258
HFD	Chow	11.016827	0.001	0.00166667
HFD +Gen	HFD+Exe	3.783856	0.005	0.0075
HFD +Gen	HFD+Gen+Exe	2.72637416	0.017	0.02125
HFD +Gen	Chow	10.6658116	0.001	0.00166667
HFD+Exe	HFD+Gen+Exe	1.95278348	0.059	0.06321429
HFD+Exe	Chow	8.42353822	0.001	0.00166667

Table S4. Weighted Unifrac pairwise-comparisons between HFD+chow, chow, HFD, HFD+Gen, HFD+Exe, and HFD+Gen+Exe after 24 weeks of six different 24-week dietary and exercise

Group 1	Group 2	Pseudo-F	p-value	q-value
HFD+chow	HFD	6.81085704	0.001	0.003
HFD+chow	HFD +Gen	7.76190527	0.001	0.003
HFD+chow	HFD+Exe	5.86103833	0.003	0.00642857
HFD+chow	HFD+Gen+Exe	4.76080063	0.002	0.005
HFD+chow	Chow	2.37328375	0.074	0.0925
HFD	HFD +Gen	2.33968388	0.07	0.0925
HFD	HFD+Exe	1.85529656	0.111	0.12807692
HFD	HFD+Gen+Exe	1.0615309	0.344	0.344
HFD	Chow	7.23363861	0.001	0.003
HFD +Gen	HFD+Exe	3.34333991	0.018	0.027
HFD +Gen	HFD+Gen+Exe	3.34990047	0.018	0.027
HFD +Gen	Chow	10.5353498	0.001	0.003
HFD+Exe	HFD+Gen+Exe	1.35804993	0.228	0.24428571
HFD+Exe	Chow	4.33439303	0.005	0.009375
